# Management of Incidental Peri-Diaphragmatic Cyst During Bariatric Surgery: A Case Report and Review of the Literature

**DOI:** 10.7759/cureus.100268

**Published:** 2025-12-28

**Authors:** Amal M Alhabib, Mohammad D Alghamdi

**Affiliations:** 1 Bariatrics and Metabolic Disorders Center, Dammam Medical Complex, Dammam Health Network, Dammam, SAU

**Keywords:** bariatric surgery, bronchogenic cysts, combination technique, incidental diagnosis, laparoscopic sleeve gastrectomy

## Abstract

Bronchogenic cysts are rare congenital anomalies that originate from the foregut and are often incidentally detected in adults during imaging studies. While they are typically asymptomatic, they can pose significant challenges during surgical interventions, particularly in patients undergoing bariatric surgery. We report a case of a 49-year-old woman with morbid obesity (BMI = 40 kg/m^2^) who underwent a preoperative evaluation for bariatric surgery. During routine ultrasonography, a 4 cm anechoic retrogastric cyst was identified, initially thought to represent a pancreatic pseudocyst. Computed tomography (CT) and magnetic resonance imaging (MRI) of the abdomen confirmed that the cyst originated from the right crus, compressing rather than arising from the pancreas. We therefore opted for a diagnostic laparoscopy to determine the origin of the cyst and manage it if feasible, along with a vertical sleeve gastrectomy to address obesity.

The surgical procedure involved gaining access to the cyst through the lesser sac after division of the greater omentum. The cyst was excised en bloc, and a vertical sleeve gastrectomy was performed over a 36F bougie. Histology demonstrated that the examined sections revealed a cyst lined by respiratory-type epithelium. The cyst wall also contained smooth muscle bundles, supporting the possibility of a bronchogenic cyst. The patient tolerated oral intake, ambulated with minimal discomfort, and was discharged with an uncomplicated postoperative course. The report highlights the importance of thorough preoperative evaluation and appropriate management of incidental findings during bariatric surgery.

Our experience demonstrates that a comprehensive preoperative assessment is vital in bariatric patients, as it allows for the identification and management of incidental findings such as bronchogenic cysts. A combined surgical approach can enable successful outcomes without delaying the planned bariatric intervention.

## Introduction

Bronchogenic cysts, first reported in 1859, are rare congenital anomalies that originate from the foregut as a result of the budding of the bronchial tree during fetal development [1]. Although commonly diagnosed in childhood, they are also observed in adults, with a higher prevalence among males [2]. These cysts are typically found in the mediastinum but can also appear below the diaphragm, including occasionally in the retroperitoneum around the left adrenal gland, and may originate from the left crus. Bronchogenic cysts are often asymptomatic and incidentally detected during imaging; however, symptoms can arise if the cyst becomes complicated by infection, bleeding, or local compression [3,4]. Histologically, a bronchogenic cyst contains normal bronchial structures, including a respiratory-type epithelial lining, hyaline cartilage, and smooth muscle [5,6]. While malignant transformation is extremely rare, sporadic cases have been reported [7,8]. Management typically involves surgical intervention for definitive diagnosis, symptomatic relief, and complication prevention, with complete resection generally yielding favorable outcomes [9].

In the context of bariatric surgery, the increasing use of comprehensive preoperative imaging has frequently led to the incidental discovery of such lesions. The presence of an incidentaloma, particularly one located near the planned surgical field, can significantly impact surgical planning and requires careful decision-making regarding its management. This involves assessing the lesion’s nature and its potential to interfere with the bariatric procedure, thereby prompting consideration of a combined or staged surgical approach to optimize patient safety and overall outcomes. This case report describes the incidental discovery, diagnostic workup, and integrated surgical management of a bronchogenic cyst encountered during bariatric surgery, demonstrating how a combined approach can successfully address this rare condition within the bariatric setting.

## Case presentation

**Figure 1:**
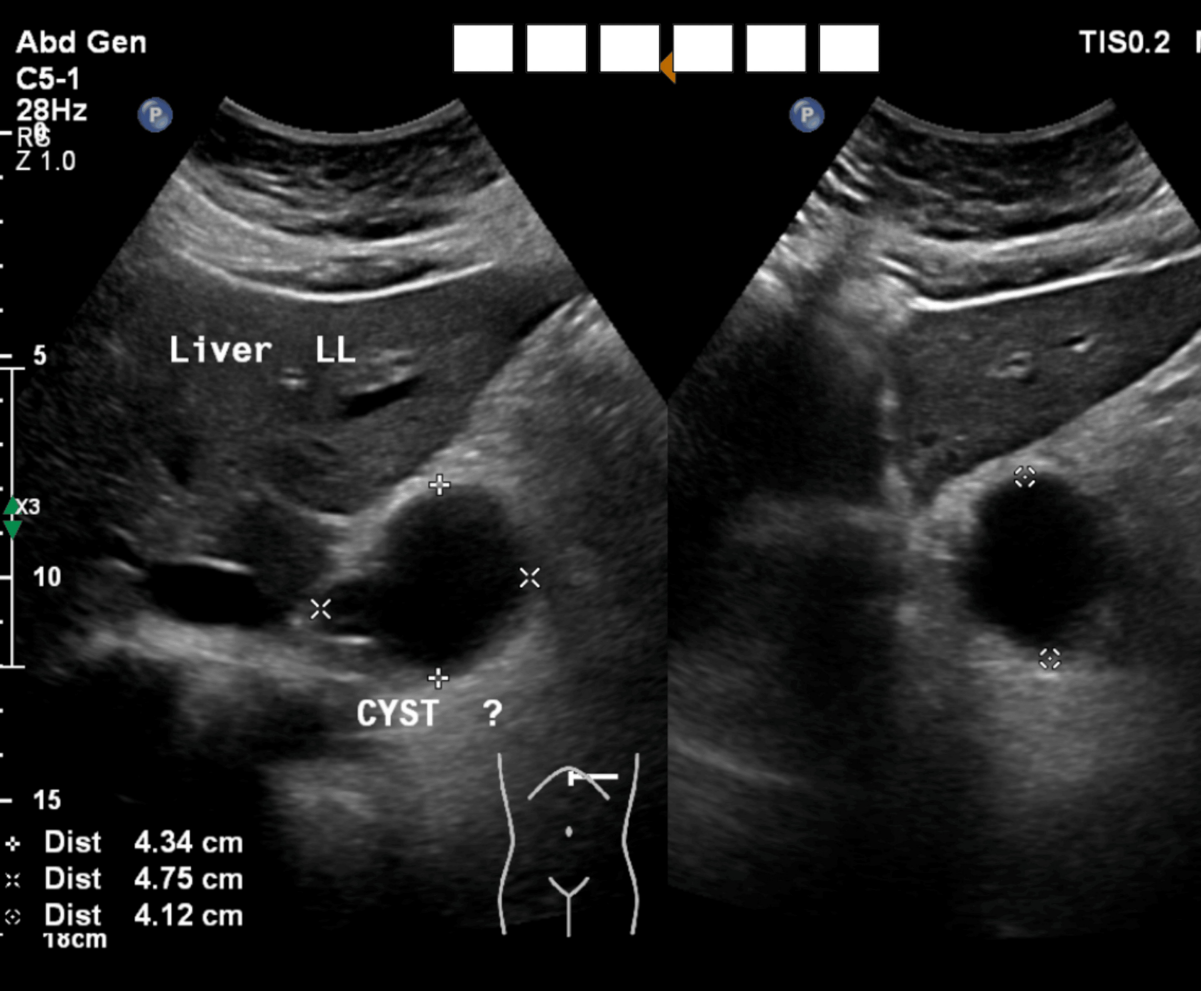
Ultrasonography findings A rounded anechoic cystic lesion was identified in the midline upper abdomen, lateral to the pancreatic head, and in the subhepatic region. The lesion measured 4.3 × 4.7 × 4.1 cm and demonstrated no internal vascularity or calcification. These findings were considered most consistent with a pancreatic pseudocyst

A 49-year-old female with morbid obesity (BMI 40 kg/m^2^) and no other chronic medical conditions had previously failed lifestyle-based weight control measures. She was undergoing a preoperative evaluation for bariatric surgery when a 4 cm anechoic retrogastric cyst was identified on ultrasonography (Figure 1).

**Figure 2:**
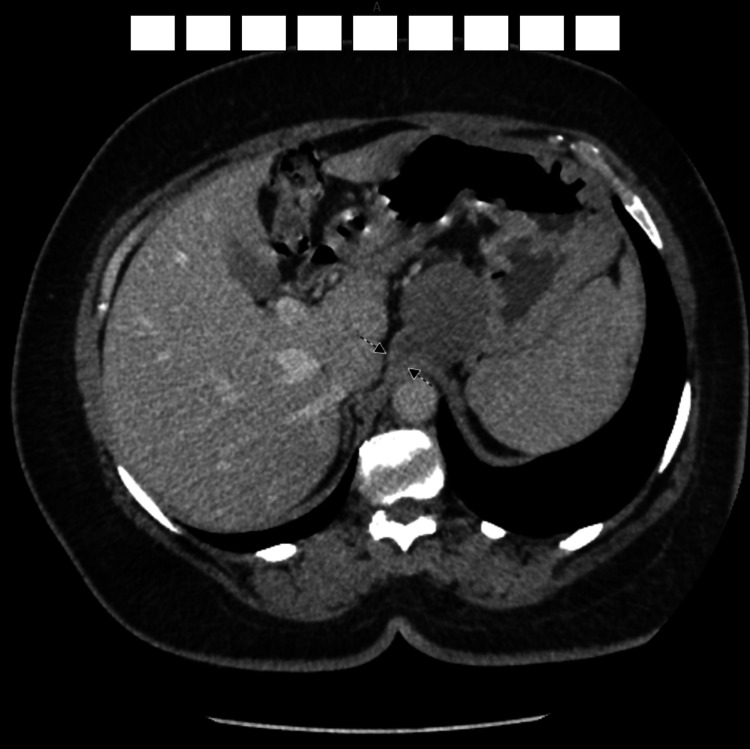
CT scan The aforementioned findings are suggestive of a foregut duplication cyst, such as a bronchogenic cyst arising from the diaphragmatic crus. A pseudocyst is another less likely possibility in the presence of a history of prior pancreatitis Black arrows pointing to the possible origin of the cyst (right crus) CT: computed tomography

**Figure 3:**
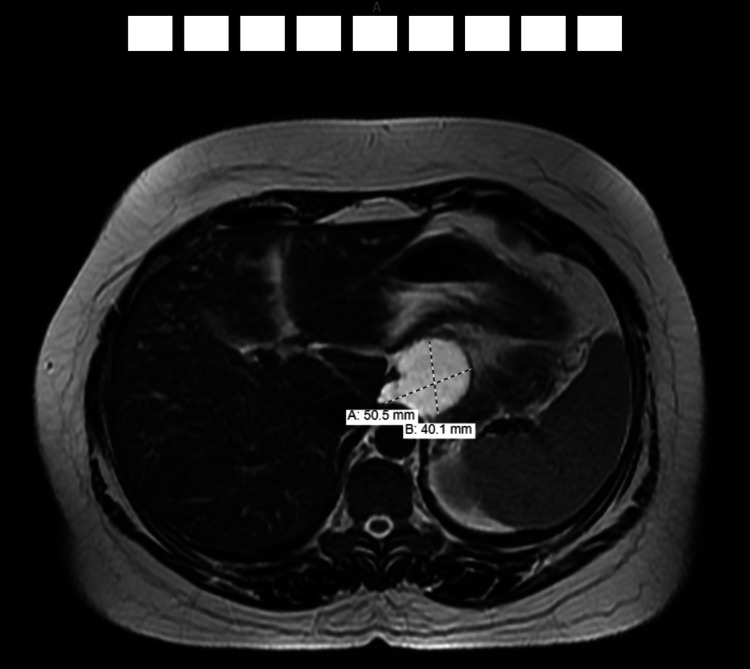
MRI findings MRI confirms the presence of a well-marginated 5 cm cyst in the left upper abdomen, likely arising from the right diaphragmatic crus as described above. It seems to be displacing the pancreas rather than arising from it. Again, differential diagnosis includes a foregut duplication cyst or a bronchogenic cyst, among others MRI: magnetic resonance imaging

Due to its retrogastric location, a pancreatic pseudocyst was initially suspected. However, normal amylase and lipase levels, demonstrating no evidence of chronic pancreatitis, along with further imaging via CT and MRI, clarified that the cyst originated from the right crus and merely compressed the pancreas. The presumptive diagnosis was subsequently established as a bronchogenic cyst, with a duplication cyst considered a less likely differential (Figures 2, 3).

We opted to perform a combined laparoscopic approach to resect the cyst while simultaneously carrying out the intended bariatric procedure, namely a vertical sleeve gastrectomy.

**Figure 4:**
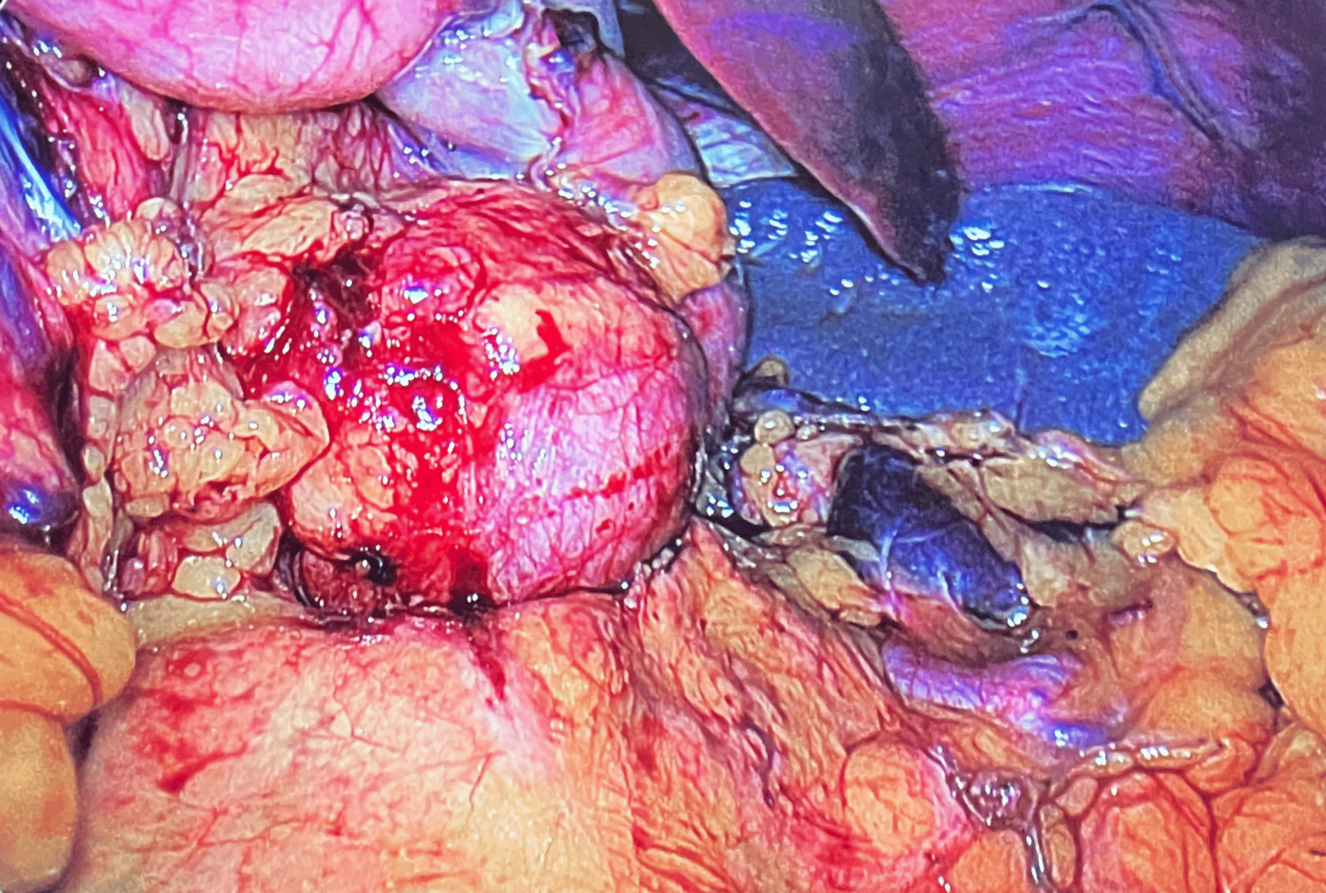
Peri-diaphragmatic cyst

**Figure 5:**
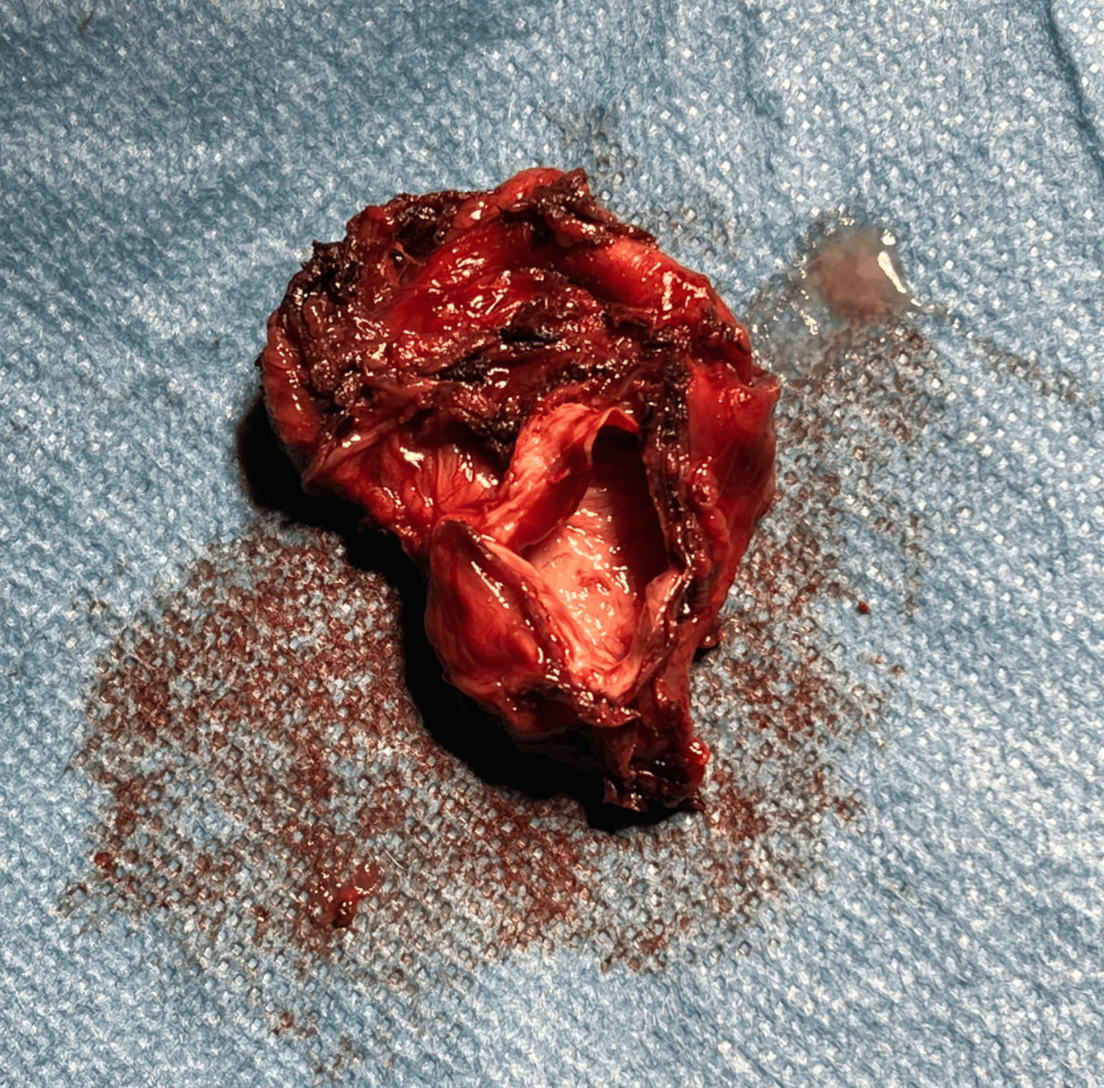
Resected peri-diaphragmatic cyst with mucus content

The procedure began with division of the greater omentum, thereby gaining access to the cyst within the lesser sac. Once the sac was fully exposed, it became clear that the cyst was amenable to complete excision (Figure 4). A vertical sleeve gastrectomy was performed over a 36 French calibration tube. The cyst originated from the junction of the right and left crura and was carefully dissected and resected en bloc. The cyst was inadvertently entered during dissection and contained thick mucoid material. It had a thick brown capsule with a smooth internal lining (Figure 5).

Microscopic examination revealed a cyst lined by respiratory-type epithelium. The cyst wall contained bundles of smooth muscle, consistent with a bronchogenic cyst, which is a benign entity with a very low risk of malignant transformation. The patient had an uncomplicated recovery, tolerated oral intake, and was followed on an outpatient basis with a normal expected postoperative course.

This report highlights the importance of a thorough preoperative evaluation in bariatric surgery patients to identify and manage incidental findings such as bronchogenic cysts. Multidisciplinary collaboration is crucial in making informed decisions regarding the optimal management approach for these rare anomalies. By sharing this case, we hope to demonstrate that a combined approach to managing an incidental finding alongside a bariatric procedure is feasible and more convenient for the patient.

## Discussion

The presence of a bronchogenic cyst, although rare, demonstrates that even asymptomatic patients can harbor significant incidental findings that may necessitate a combined surgical approach. Previous reports have documented the deferral of bariatric procedures due to concerns regarding the nature of the cyst and the potential consequences of resecting an uncharacterized lesion. However, our experience suggests that addressing both conditions simultaneously can be both practical and advantageous. In recent years, incidental findings during bariatric surgery have been increasingly reported in the literature, representing a small but clinically relevant percentage of cases. Studies have shown that incidental findings, particularly on preoperative imaging, can range from benign lesions to more concerning pathologies such as gastrointestinal stromal tumors (GISTs). For example, recent data from a single-institution study identified incidentalomas in approximately 2% of bariatric patients, most commonly involving the stomach, liver, and gallbladder. GISTs, although rare, have been among the most clinically significant incidental findings encountered during these procedures [10,11].

The decision to proceed with or postpone bariatric surgery is often based on whether the incidental finding might interfere with surgical outcomes. A case has been reported in which the surgeon opted for cyst resection alone and delayed the bariatric procedure due to the anticipated high risk [12]. In contrast, incidental findings that do not affect the planned procedure generally allow the surgery to continue as intended. When an incidental finding does require surgical attention, laparoscopic techniques have proven particularly valuable, enabling precise management of distant or difficult-to-reach lesions. Our approach emphasizes the importance of tailoring surgical interventions to ensure the safety and success of the bariatric procedure. For example, initiating laparoscopic exploration by creating a window through the greater curvature is recommended, as this technique provides optimal exposure and easy access to the cyst, allowing the bariatric intervention to proceed smoothly. By performing the vertical sleeve gastrectomy first, we controlled the area around the cyst, facilitating a safe, en bloc resection with minimal intraoperative risk.

Combining procedures in this manner has significant effects on patient satisfaction and well-being. By addressing both the primary and incidental findings in a single operation, we reduce the risks linked to multiple surgeries, shorten recovery time, and prevent potential delays in treatment. This comprehensive approach enhances the surgical experience and outcomes, promoting higher patient satisfaction by removing the need for additional interventions.

In summary, the management of incidental findings such as bronchogenic cysts during bariatric surgery requires meticulous planning and collaboration among multiple specialties. Sharing this case underscores the need for bariatric surgeons to remain vigilant and adaptable when confronted with unexpected discoveries. By recognizing and addressing these rare anomalies and utilizing the advantages of laparoscopy for distant incidental findings, we can optimize surgical outcomes and enhance overall patient well-being.

## Conclusions

A comprehensive preoperative evaluation is essential for patients undergoing bariatric surgery to identify incidentalomas, such as bronchogenic cysts, and formulate an appropriate surgical strategy. Early identification enables the possibility of a combined surgical approach to address both the bariatric procedure and any incidental findings, thereby reducing the need for subsequent interventions, improving efficiency, and shortening recovery time. When incidentalomas are discovered intraoperatively, the surgical team must assess their potential impact on the planned procedure. If the incidental finding does not compromise the bariatric surgery, the operation may proceed as intended. Conversely, if there is a significant risk, the surgical approach may need to be adjusted to safely manage the incidentaloma. By anticipating such scenarios, surgeons can enhance patient outcomes and ensure a more streamlined, patient-centered experience.
